# Atrial Tachycardia with Cycle Length Alternans

**DOI:** 10.19102/icrm.2023.14052

**Published:** 2023-05-15

**Authors:** Ahmet Korkmaz, Özcan Özeke, Serkan Cay, Firat Ozcan, Meryem Kara, Elif Hande Ozcan Cetin, Nur Beton, Atik Aksoy, Halenur Saribas, Idriz Merovci, Can Demirhan, Dursun Aras, Serkan Topaloglu

**Affiliations:** ^1^Department of Cardiology, University of Health Sciences, Ankara City Hospital, Ankara, Turkey; ^2^Department of Cardiology, University Clinical Center of Kosovo, Prishtina, Kosovo; ^3^Department of Cardiology, Istanbul Medipol University, Istanbul, Turkey

**Keywords:** Atrial tachycardia, double tachycardia, dual loop, multiple loops

## Abstract

Tachycardia-induced tachycardia, or so-called double tachycardia, appears to be a relatively rare condition. The underlying mechanism for stable beat-to-beat cycle length variability (alternans) in atrial tachycardia has been sparsely reported.

## Case presentation

**Figure 1:**
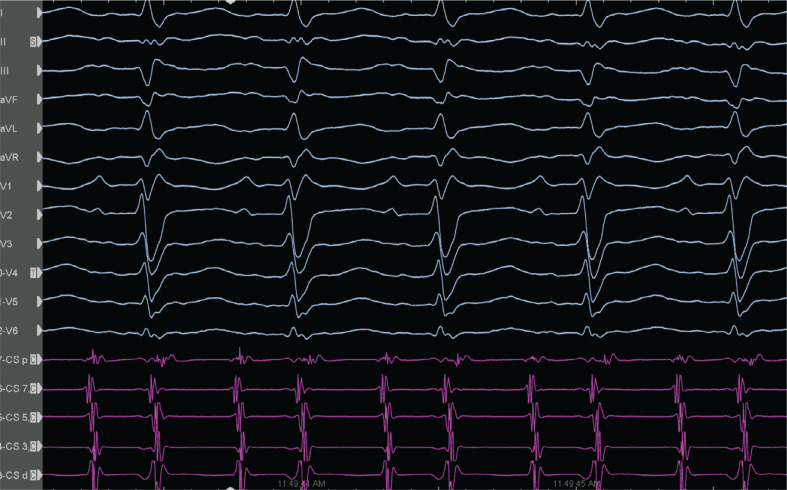
Electrocardiography showing the supraventricular tachycardia with cycle length alternans.

A 65-year-old male patient without a history of previous electrophysiological study (EPS), ablation, or cardiac operation presented with incessant narrow complex tachycardia (NCT) and was referred for an EPS. Three years earlier, he had undergone percutaneous transluminal coronary angioplasty with stenting for single-vessel coronary artery disease. The initial cycle length (CL) was 290 ms. Three-dimensional electroanatomic mapping (EAM) (CARTO III software version 7, Carto Prime; Biosense Webster, Diamond Bar, CA, USA) was performed in the right and left atria (RA and LA, respectively) using a PentaRay catheter (Biosense Webster). An attempt was made to entrain atrial flutter by pacing from the LA, which converted it to atrial tachycardia (AT) with alternating CLs of 290 ms (CL-1) and 230 ms (CL-2) **(Figure 1)**. What are the possible mechanisms of these alternating CLs?

## Discussion

The differential diagnosis for NCT with alternating CL includes a regularly irregular AT,^1–6^ orthodromic atrioventricular (AV) reciprocating tachycardia (AVRT) using 2 distinct retrograde accessory pathways,^6–15^ and atypical AV nodal re-entry tachycardia (AVNRT) with multiple retrograde AV nodal pathways.^16–25^ Multiple supraventricular tachycardias with multiple re-entry circuits are also relatively common in a patient during EPS. It has been reported that some AVNRTs might reset the AT focus when anterograde conduction is over the faster or slower pathway.^16,26^ The first step in analyzing tachycardia is finding the P-waves. AVRT and AVNRT can easily be ruled out by the morphology and activation sequence of the P-wave (indicating a craniocaudal sequence).^19^ Furthermore, when atrial CL variability precedes ventricular CL variability, AT is commonly the mechanism underlying the NCT.^13^ In the current case, atrial CL variability preceded ventricular CL variability and the positive P-wave polarity made the diagnosis of AT easy.

Stable beat-to-beat CL variability (CL alternans) in AT has been sparsely reported,^1–5,27^ especially in complicated tachycardia.^1,2,4,16,27–29^ Recently, Takigawa et al. found that 16.7% of cases had CL variability in their multiple-loop AT group, and this CL variability was more frequently observed in non-anatomic macro–re-entrant ATs.^30^ Frontera et al. determined that re-entrant ATs have multiple sequential isthmuses of slow conduction, serving as sequential isthmuses rather than a single isthmus.^29^ Sometimes, a single tachycardia may initiate another or occur concurrently with the latter. While the faster index AT is running, the index AT repetitively resets the second longer circuit in each beat.^31^ One AT focus is allowed to depolarize when the other AT conduction occurs over the slower isthmus conduction, and, if these tachycardias have similar intra-atrial activation sequences, the situation can be very challenging. Some complex patterns may involve an atrial activation duration (AAD), which is longer than the tachycardia CL, which makes maps difficult to interpret.^32^ Therefore, we can summarize the possibilities as follows: (1) dual loop with separated isthmus, (2) dual loop with the same shared isthmus, (3) dual ATs consisting of a focal AT and re-entrant other AT, or (4) an AT focus with dual firing when the AAD lasts longer than the length of the tachycardia cycle.^27,32^ Of course, the distinct recording of alternate fractionated potentials by EAM with high-density catheters is important to evaluate the complex atrial substrate.^5,27^ Additionally, the threshold for stable CL criteria or parallel mapping during the EAM can be adjusted to focus on the target CL, resulting in separated and accurate maps for long and short CLs, respectively.^27^

**Figure 2:**
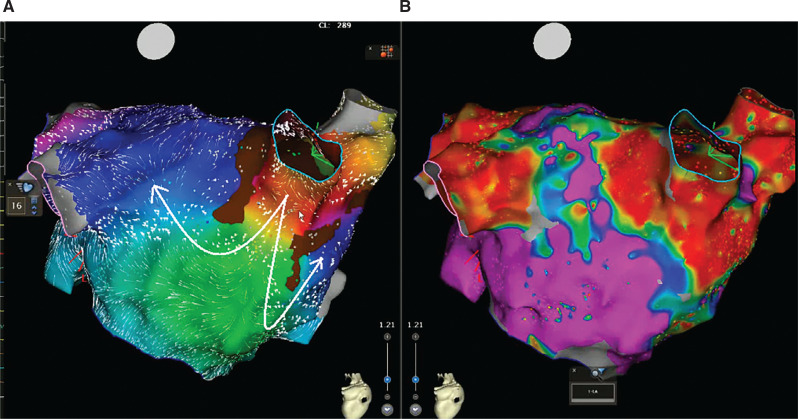
The electroanatomic map of the left atrium from the posterior view with **(A)** coherent mapping and **(B)** voltage mapping shows a focal site of earliest activation from the right inferior pulmonary vein spreading in all directions after posterior exit.

**Figure 3:**
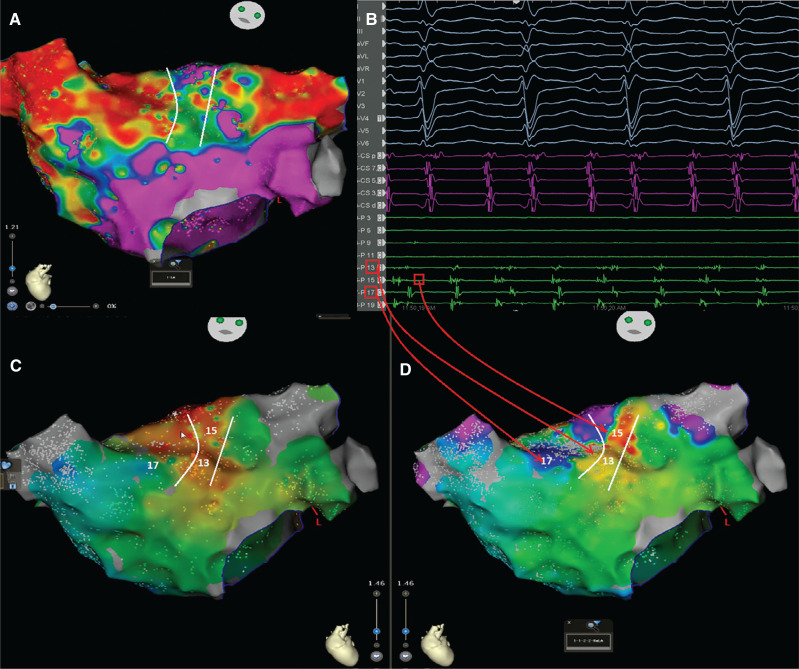
The electroanatomic map of the left atrium from the anterior view with **(A)** voltage mapping and **(B)** intracardiac electrography. Parallel mapping for **(C)** the CL-2 atrial tachycardia and **(D)** the alternating tachycardia. The change in the intracardiac electrograms preceded the tachycardia cycle length, particularly in the proximal coronary sinus (see channels 13 and 17). Note that the longer conduction time depicted by fragmentation in the isthmus (area 13) was associated with lateness, causing color changes in area 17 and suggesting the competing activation between both tachycardias and passive circuits.

**Figure 4:**
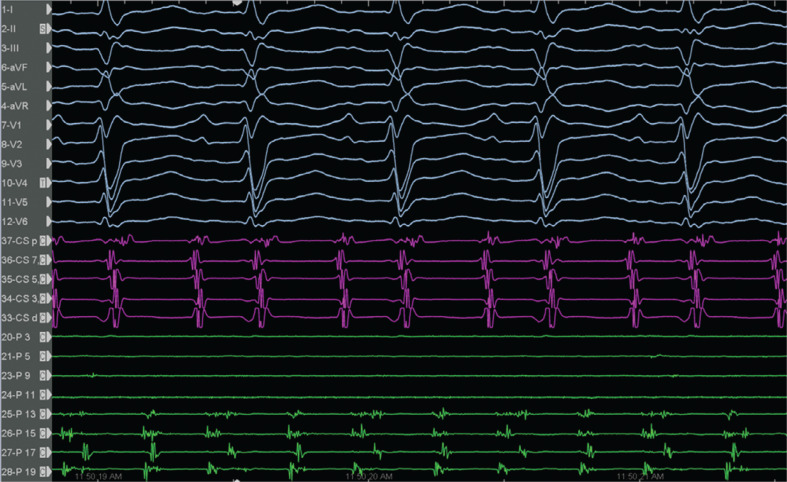
Note that the alternating of the double potentials and single or fragmented potentials corresponded to the cycle length alternans at a fixed location.

**Figure 5:**
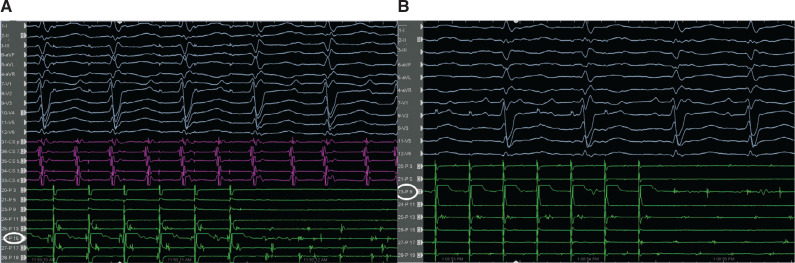
**A and B:** The left atrial anteroseptal roof site was considered to be in the re-entrant circuit based on the short post-pacing intervals.

In the current case, during EAM, the window of interest (WOI) of the slower CL-1 tachycardia was explored and an activation sequence consistent with a focal AT (automatic or triggered foci or micro–re-entry) of the right inferior pulmonary vein (RIPV) was noticed **(Figure 2, Videos 1 and 2)**. The simultaneous activation of the RIPV and the anteroseptal side of the roof (note the red area in **Video 1**) raised the possibility of the AAD lasting longer than the length of the tachycardia cycle.^32^ We attempted an entrainment maneuver to clarify the mechanism of the tachycardia originating from the RIPV (automatic or triggered foci or micro–re-entry); however, it converted to the CL-1 AT and to an AT with alternating CLs of 290 ms (CL-1) and 230 ms (CL-2) **(Figure 1)**. As the EAM system offers an opportunity for simultaneous activation mappings under the 2 different CLs using a parallel mapping system, a second EAM using the parallel mapping function was subsequently constructed for the shorter CL-2 tachycardia (260 ms), and, again, the entire CL could be mapped around the left atrial roof **(Video 3)**. The change in the intracardiac electrograms (EGMs) preceded the tachycardia CL (**Figure 3**; note that the longer conduction time depicted by fragmentation in the isthmus [channel 13] is associated with lateness, causing the color changes [channel 17]). The alternating of the double potentials and single or fragmented potentials corresponding to the CL alternans at a fixed location **(Figures 3B and 4, Video 4)** can explain the AT with alternating CLs.^27^ The consistency of those post-pacing intervals in the possible isthmus from the anteroseptal roof **(Figure 5)** supported the re-entry mechanism for CL-2. As there was a narrow live corridor between the low-voltage areas **(Video 5)**, the CL alternans might have also been caused by the alternate conduction velocities between this protected valley and the slow conduction region.^5,27^ A stable and perfect timing for the recovery of conduction is considered very uncommon.^27^

On the other side, a competition for the activation of some areas (color area 17 in **Figure 3**) by AT-1 and AT-2 sequentially may have been at least partially responsible for these EGM changes. Variable fusion and wavefront collisions (WFCs) occurring at different CLs on the available intracardiac EGMs indicate the competing activations between CL-1 and CL-2 probably in passive areas (**Figure 3**; note as well the changes in EGMs and color in area 17 and the changes in reference coronary sinus [CS] EGM morphology immediately after the first 2 ablation points in **Video 6**).^31,33,34^ ATs with AAD therapy lasting longer than the tachycardia CL have been found in approximately 10% of ATs, mostly in those caused by localized r-eentry.^32^ The EGMs may be inscribed after activation along the active circuit or after the focus has been completed (**Video 1**; note the simultaneous activation of the RIPV and anteroseptal roof stressed by a dark blue color). Indeed, this is a constraint of platforms that rely on the WOI.^35^ We did not attempt multi-site entrainment to differentiate macro– versus localized re-entry; however, the WFC sites were detected at the medial side of the anteroseptal roof (area 17 in **Figure 3**).^31^

Altogether, we considered that the presence of the focal RIPV tachycardia and roof-dependent AT using the particularly left pulmonary veins might be responsible for these alternating ATs.^30,31^ The long isoelectric line between the P-waves **(Figure 1)** also supported the dual-loop re-entry with the combination of 2 non-anatomic macro–re-entrant ATs.^30^ The high-resolution EAM may facilitate tailored ablation in the most practical and vulnerable isthmuses.^27,36^ Irrigated radiofrequency ablation with a maximum energy level of 30 W was first performed in the anteroseptal roof area (see channel 13 in **Figure 3** and see **Videos 6 and 7**), and only a few ablation points changed first the CS reference EGM morphology **(Video 6)** and subsequently the CLs of the alternating tachycardias to a fixed CL-1 tachycardia **(Video 7)**. After the first ablation set, as seen in **Videos 6 and 7**, we completed the roof line, and the post-pacing interval was no longer in the circuit in the roof area **(Video 8)**. Finally, CL-1 tachycardia termination was easily achieved by isolation of the RIPV.^33,37^ The patient was discharged from the hospital the following day after his procedure, and his follow-up has been uneventful for 2 months since the first postoperative visit. It is important to be alert to the possibility of double tachycardia during the ablation procedure as the switch from a certain tachycardia to another creates confusion.^27,38^

## Figures and Tables

**Video 1:** Propagation mapping of the alternated tachycardia. Note the simultaneous activation of the right inferior pulmonary vein, and anteroseptal roof stressed by a dark blue color.

**Video 2:** Coherent mapping of the CL-1 tachycardia.

**Video 3:** Propagation mapping of the CL-2 tachycardia by parallel mapping.

**Video 4:** Note that the alternating of the double potentials and single or fragmented potentials corresponded to the cycle length alternans at a fixed location in the suspected isthmus.

**Video 5:** Note that the alternating of the double potentials and single or fragmented potentials corresponded to the cycle length alternans at a fixed location in a more lateral narrow corridor area.

**Video 6:** The change in the reference coronary sinus electrogram morphology by limited/pragmatic ablation of the anteroseptal isthmus point.

**Video 7:** The conversion of the alternated tachycardia to the CL-1 tachycardia by limited/pragmatic ablation of anteroseptal isthmus point.

**Video 8:** Entrainment attempts at the posterior roof after lesion set completion for the CL-2 tachycardia shows out of the circuit anymore.
